# In lumbosacral plexus injuries can we identify indicators that predict spontaneous recovery or the need for surgical treatment? Results from a clinical study on 72 patients

**DOI:** 10.1186/1749-7221-9-1

**Published:** 2014-01-11

**Authors:** Debora Garozzo, Gianluca Zollino, Stefano Ferraresi

**Affiliations:** 1 Department of Neurosurgery, Ospedale S. Maria della Misericordia, Viale Tre Martiri 140, 45100 Rovigo, Italy

**Keywords:** Lumbosacral plexus, Nerve injury, Root avulsions, Pelvic trauma, Sacroiliac joint separation, Sacral fracture

## Abstract

**Background:**

Post-traumatic lumbosacral plexus injuries seem to be rare events, spontaneously recovering in high percentage: as surgery is often challenging and results in poor outcome, many Authors have advocated conservative treatment only. Nevertheless surgery should not be ruled out: in invalidating injuries, it can restore basic function in the lower extremities.

Therefore, it might be necessary to establish guidelines for the management and the indication to surgery in such cases.

This study aims to identify indicators predicting spontaneous recovery or the need for surgery.

**Method:**

The clinical and radiological data of 72 patients with a post-traumatic lumbosacral plexus injury were reviewed. A follow up equal or superior to 3 years is available in 42 cases.

**Results:**

Lumbosacral plexus injuries mostly occurred during road accidents. The incidence of associated lesions was relevant: bone injuries were found in 85% of patients, internal lesions in 30% and vascular injuries in 8%.

Lumbosacral trunk and sacral plexus palsies were the most frequent injury patterns.

Root avulsions were revealed in 23% of cases and only in sacral plexus and complete lumbosacral plexus injuries: L5 and S1 were the roots more prone to avulsions.

About 70% of cases recovered spontaneously, mostly in 18 months. Spontaneous recovery was the rule in lumbar plexus and lumbosacral trunk injuries (where root avulsions never occurred) or in sacral and complete lumbosacral plexus palsies due to compression injuries.

The causative mechanism correlated with the injury pattern, the associated bone injury being often predictive of the severity of the nerve injury.

Lumbosacral plexus injuries occurred in car crashes were generally associated with fractures causing compression on the nerves, thus resulting in injuries often amenable of spontaneous recovery.

Motorcycle accidents implied high kinetic energy traumas where traction played an important role, as the high percentage of sacroiliac joint separations demonstrated (found in more than 50% of cases and always associated to root avulsions).

Loss of sphincteral control and excruciating leg pain were also invariably associated with avulsions.

**Conclusions:**

Clinical and radiological data can help to predict the occurrence of spontaneous recovery or the need for surgery in post-traumatic lumbosacral plexus injuries.

## Background

The lumbosacral plexus was illustrated by the Italian anatomist Giulio Casserio in 1632 [1] but the first description of a post-traumatic injury only dates back to approximately 50 years ago [2].

Lumbosacral plexus (LSP) injuries are considered rare events and their spontaneous recovery occurs in high percentage [3-6]: on the other hand, surgery is often demanding and implies multidisciplinary cooperation, whereas outcome is frequently poor. Therefore, some Authors advocate only conservative treatment [5].

Yet, in cases of severe, devastating injuries with no chances of spontaneous recovery, the role of surgery should not be denied as it could offer undoubted advantages to patients otherwise destined to permanent disability [7].

It can be clearly inferred that there is a need to establish precise guidelines concerning the management and timing for surgery in these lesions, ruling out those patients amenable of a spontaneous recovery.

The aim of this study was to identify indicators that could help to predict the occurrence of spontaneous recovery or the need for surgical treatment.

## Methods

The present study was a retrospective review of a series of 72 patients with a post-traumatic LSP injury. They were referred to the Authors’ observation several months or even years after the injury in the period between January 1991 and May 2009.

The Authors collected and analysed the data regarding:

a) Gender and age incidence

b) Causative events

c) Timing of diagnosis

d) Association with other injuries

e) Incidence of bilateral injuries

f) Injury patterns

g) Incidence of root avulsions

h) Natural history

a) ***Gender and age incidence***

The series consist of 60 males and 12 females, including two children (a 4 year old boy and a 18 month old girl). Patients’ age ranged from 18 months to 68 years, with no peak of age.

b) ***Causative events***

LSP injuries mostly occurred in road accidents: car crashes in 59% of cases and motorcycle accidents in about 20%.

A detailed description of the causative events is given on Table 1.

c) ***Timing of diagnosis***

The nerve injury was diagnosed during the early assessment after the trauma in 30% of cases, about one month later in 20%, within 3 months in 25%, within 6–8 months in 10% of cases. In 5% of patients the nerve injury was diagnosed one year after the traumatic event.

d) ***Associated injuries***

The patients described in this series were often multi-traumatized but for “associated injuries” here we deal only with the injuries strictly correlated to the LPS injury: bone injuries of the pelvic ring and proximal femoral fractures, internal and vascular injuries.

**Figure 1:**
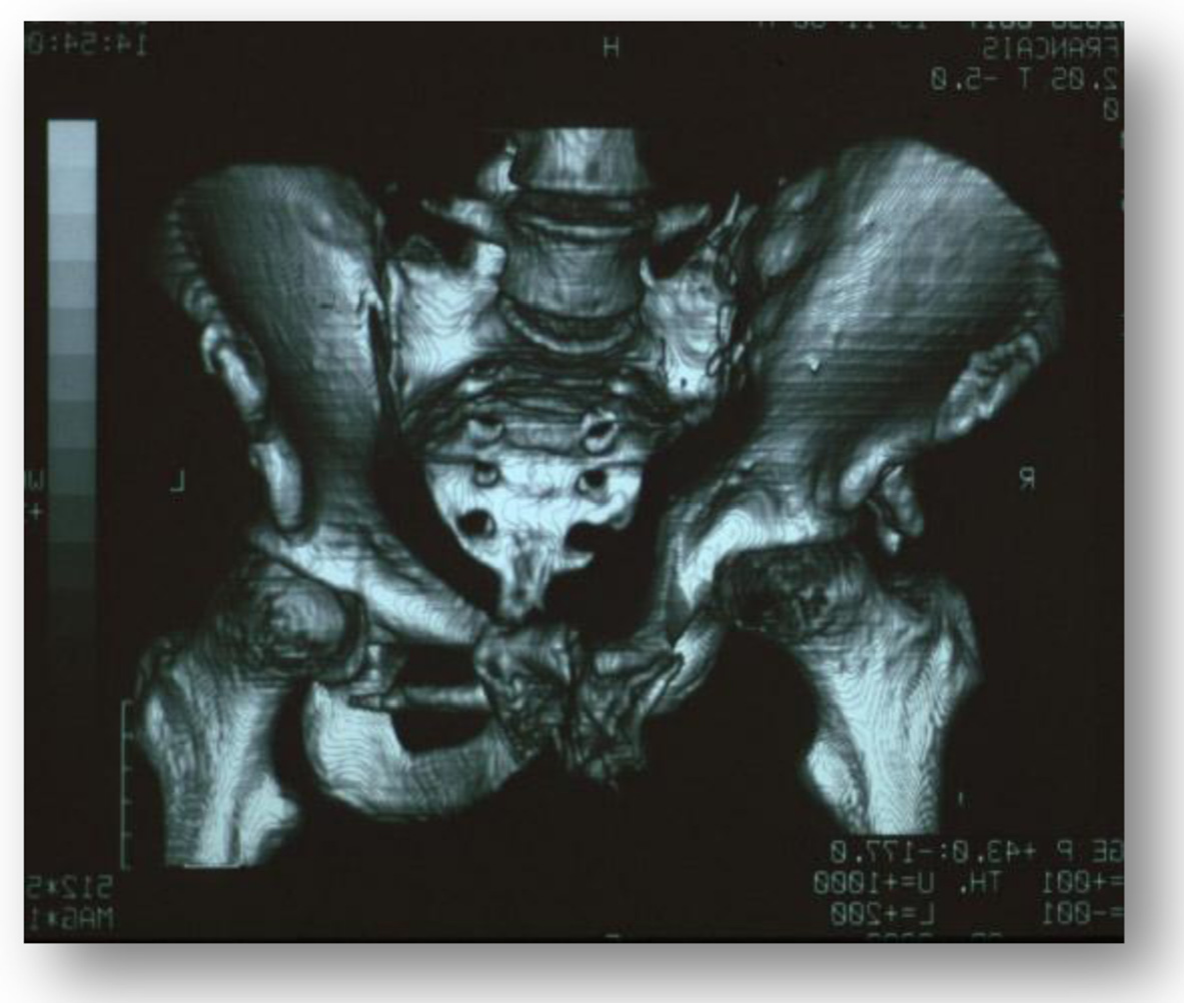
Acetabular fracture.

**Figure 2:**
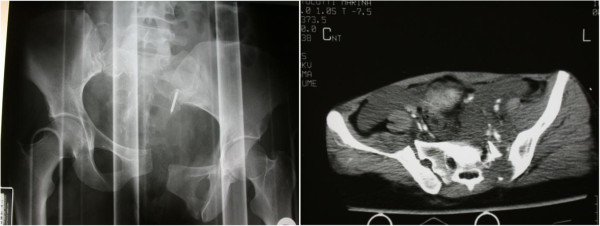
Sacroiliac joint separation.

Bone injuries were found in 83% of cases. Every type of pelvic fracture could be found (including comminuted fractures of the pelvic ring); iliac-ischiopubic fractures, acetabular fractures (Figure 1), sacroiliac joint separations (Figure 2) and sacral fractures were the most frequent injuries.

Internal injuries were present in about 30% of patients. Extraperitoneal bladder ruptures with a clinical presentation of hematuria, abdominal pain and/or abdominal swelling and requiring urgent surgery were the most common internal injuries (found in more than 50% of cases): they could be caused by direct penetration of a bone fragment in ischiopubic or sinphysis fractures or due to rupture or stretching injury of the pubo-prosthatic ligament. Bladder injuries were more likely to occur when the organ was full.

Intestinal perforation generally occurred in the terminal part of the sigmoid colon or rectum.

Vascular injuries were found in about 8% of cases: in these patients a retroperitoneal haemorrhage due to bleeding of the gluteal (2 cases) or iliac vein (2 cases) or artery (2 cases) required urgent embolization. In 30% of cases nerve injuries were associated with both bone and internal or vascular injuries.

e) ***Incidence of bilateral injuries***

A bilateral palsy of the lumbosacral plexus occurred in 5,5% of cases (4 patients): 1 bilateral lumbar plexus injury, 2 bilateral sacral palsies, 1 complete lumbosacral palsy associated with a sacral palsy.

f) ***Clinical patterns***

Patients were always evaluated clinically and by electrodiagnostic and radiological studies.

Clinical assessment was focused to reveal muscles weakness and sensory disturbances as well as the presence of pain. The Medical Research Council Grading system was used to classify muscle strength [8].

**Table 1:** Causative mechanisms in lumbosacral plexus injuries

	**Car crash**	**Motorcycle crash**	**Other road accidents**	**Work accident (different from falling from on high)**	**Sport accident**	**Accidental falling from above (including work accident)**	**Other**
**Detailed description of the causal mechanisms**	Frontal impact against another veicle (car, van, track, motorcycle): **9** cases	Frontal impact against another veicle (car): **6** cases	**3** patients (2 small children) run down by a car	**1** patient° crushed by a silo falling from on high on a ship	**1** ski accident (hitting a pole during downhill)	**1** patient falling from the fourth floor	**1** patient run down by an earthwork and hit by a slab of asphalt
	Frontal impact against the guardrail: **2** cases or a wall: **1** case or a gate: **2** cases	Bus/track overriding the motorcycle: **2** cases	**1** patient crushed by a car against a gate	**1** patient whose foot was trapped and pulled by a reaper machine	**1** watermotorcycle accident	**1** Pt. fell off a ladder, lied on the floor on his left side for several hrs before rescue (nervous ischemia?)	
	Frontal impact against a tree lining the road: **4** cases				**1** patient slipping and falling down during a basketball match	**2** patients falling from a scaffold (work accident)	
	Driving off the road: **8** cases						
	No clear car accident dynamics:**17** cases	No clear accident dynamics: **7** cases					
**Total number of patients**	43*	15*	4	2	3	4*	1

*patient with associated brachial plexus palsy °patient with bilateral lumbosacral plexus palsy.

A thorough examination of the hip girdle and leg enabled to classify the injury pattern. The superior gluteal nerve and the posterior cutaneous nerve of the thigh are two nerves whose function was carefully assessed to determine the level of the injury. The superior gluteal nerve emerges from the L5 spinal nerve or the lumbosacral trunk and from the S1 spinal nerve in the proximal part of the lumbosacral plexus: abolished activity in glutei muscles impairs pelvic stability resulting in a positive Trendelemburg test. The posterior cutaneous nerve of the thigh forms at the lateral part of the sacral plexus as the sacral spinal nerves merge to form the sciatic nerve: its intact function (sensation on the dorsal aspect of the thigh) with abolished activity in harmstrings and muscles distal to the knee indicates a proximal sciatic nerve lesion rather than a sacral plexus injury.

Concerning electrodiagnostic tools, studies with H-reflex and F-response as well electromyography (EMG) were usually performed: EMG could be repeated at regular intervals to reveal possible signs of progressive reinnervation.

Radiological investigations included X-rays, angiography, CT and MRI imaging: these examinations were generally performed during the early assessment after the trauma whereas.

CT myelography and 3 dimensional MRI (3DMRI) were prescribed after avulsive injuries were suspected (mostly after referral to the Authors).

Due to the 4 bilateral palsies, 76 injuries were analysed. Based upon semeiology, 4 types of LSP injuries could be identified:

1. **A lumbar plexus injury** was the rarest pattern, found in 10% of cases (7 patients). In 6 cases (including the bilateral palsy) the clinical presentation consisted of weakness of the ilio-psoas and the quadriceps; impairment of both the femoral and the obturator nerves with palsy of the iliopsoas, quadriceps and hip adductors was found in one patient.

Femoral injuries were associated with femoral fractures in 50% of cases. In one patient X-rays revealed no bone injuries but CT detected a bulky hematoma in the psoas muscle (sport accident during a basketball match, see Table 1).

2. **A lumbosacral trunk injury** was found in about 38% of cases (29 palsies). Clinical presentation always involved the lateral contingent of the sciatic nerve (TA, ECD & EPA) plus a partial impairment of the medial contingent (TP).

A positive Trendelenburg’s sign was found in 65% of cases (19 patients).

3. **A sacral plexus injury** was the most frequent clinical presentation (42% of palsies, including 2 bilateral injuries): all the hip and leg muscles with the exception of the iliopsoas, quadriceps and hip adductors were impaired. In 8 cases sphincteral control was abolished, 3 patients also reported impairment of the erectile function.

About 1/3 of these patients complained of persisting, shooting leg pain.

4. **A complete LSP injury,** due to the involvement of all the nerves forming the plexus, was evident in 13% of patients (10 cases, 1 case in association with a sacral palsy); in 6 sphincteral control was abolished. 2/3 of these patients suffered excruciating, shooting leg pain.

g) ***Incidence of root avulsions***

**Figure 3:**
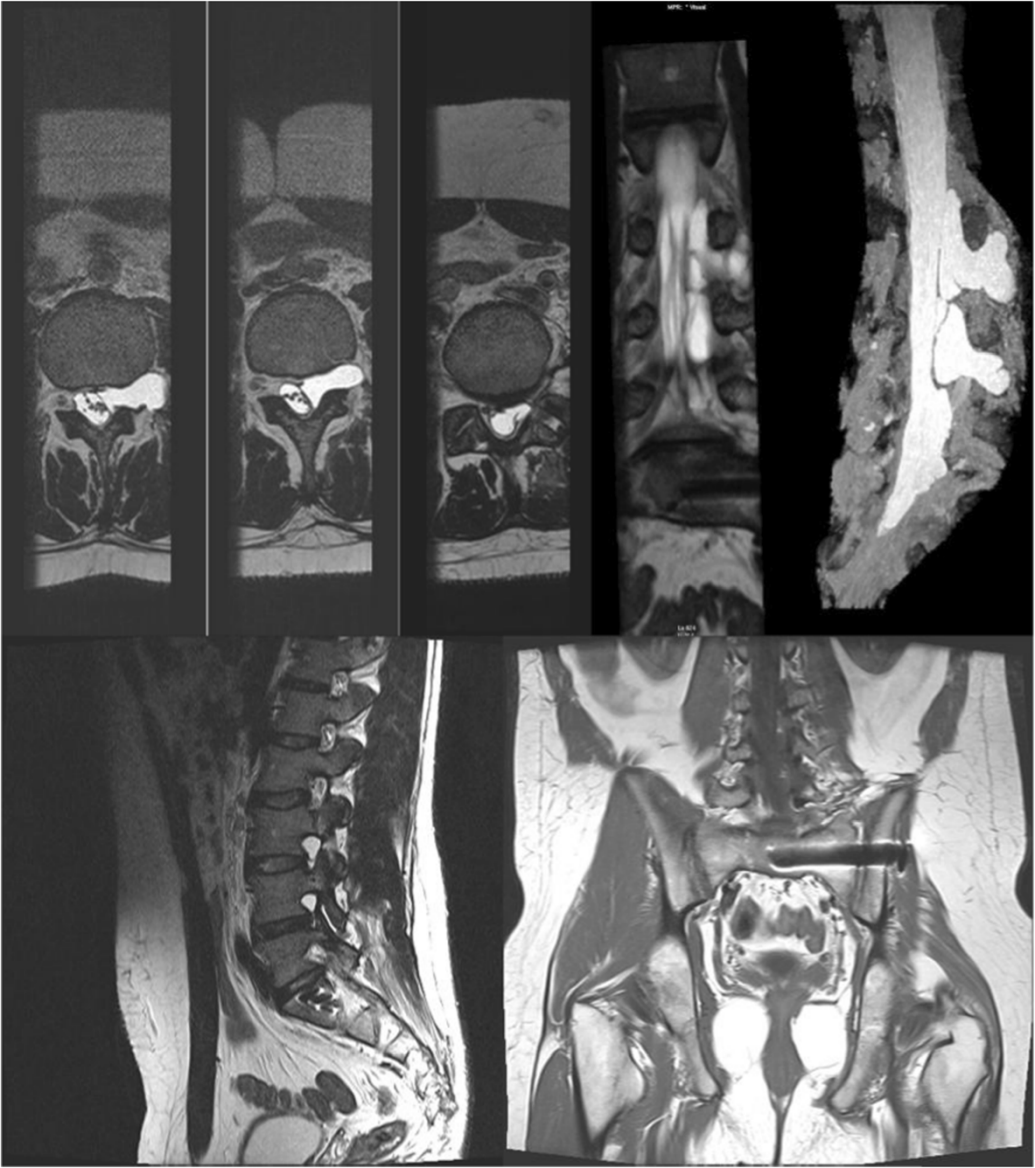
MRI study of complete LSP injury with multiple avulsions.

Avulsions were diagnosed in 23% of cases by MyeloCT and/or 3DMRI, the latter becoming the exam of first choice in our hands after 1994. Pseudomeningoceles, dural abnormalities and the absence of nerve roots inside the dural sleeves are findings that reveal the occurrence of root avulsions (Figure 3).

Root avulsions were never found in lumbar plexus or lumbosacral trunk injuries.

In sacral plexus injuries and complete lumbosacral plexus injuries, avulsions mostly occurred at L5 and S1. L4 and lower sacral root avulsions were far less frequent; L1 spinal root was never found avulsed and L2 avulsion was revealed in one case with panavulsive injury only.

h) ***Natural history***

48 patients out of 72 have a follow up study (from a minimum of 3 years).

6 patients underwent nerve surgery: radiological investigations revealed multiple avulsions and indication to surgery was straightforward. Surgery was performed between 7 months and one year after the trauma, timing of surgery depending on the referral time. Although surgical outcome is beyond the scope of this paper, Table 2 reports the clinical and radiological data, the surgical procedures and outcomes of these cases.

In 42 patients no nerve surgery was performed, the patients followed a rehabilitation treatment.

Spontaneous recovery occurred in about 70% of these cases: it was complete in 40% of cases whereas mild sequelae were left in 30% of these patients. In some cases of incomplete spontaneous recovery (4 lumbosacral trunk injuries and 4 sacral plexus palsies), posterior tibialis tendon transfers were performed to recover foot dorsiflexion.

Spontaneous recovery occurred between 3 months and 4 years from the trauma, being in most cases evident after 18 months.

Table 3 reports the clinical evolution of the patients that received conservative treatment.

**Table 2:** Nerve surgery and outcomes

**Patients’ age (years) and gender, causative mechanism**	**Injury pattern**	**Associated injuries**	**Root avulsions**	**Timing of surgery (months after injury)**	**Surgical procedure**	**Outcome**
33, M, motorcycle accident	Complete lumbosacral injury	Sacroiliac joint separation, bladder injury	L5, S1	9	Laminotomy + Henry’s approach: from the contralateral plexus sural grafts to the gluteal nerves	No result
13, M, sport accident	Complete lumbosacral injury	Sacroiliac joint separation, retroperitoneal hematoma	L3,L4,L5	12	Anterolateral extraperitoneal approach: from the contralateral obturator nerve two grafts to the femoral nerve	No result
20, F, car crash	Complete lumbosacral injury	Multiple fractures of the pelvic ring, femoral fracture	L4, L5, S1	10	Laminotomy + Henry’s approach: from the contralateral plexus sural grafts to the gluteal nerves	No result
18, M, motorcycle accident	Complete lumbosacral plexus injury	Sacroiliac joint separation, retroperitoneal hematoma	L2,L3,L4, L5, S1,S2	9	Laminotomy + anterior approach: from the contralateral plexus 2 sural grafts to the femoral nerve	No result
25, M, motorcycle accident	Complete lumbosacral palsy	Sacroiliac joint separation	L4,L5,S1	7	Laminectomy + Henry’s approach: from the contralateral plexus nerve grafts to the gluteal and sciatic nerves	No result
24, M, car crash	Sacral plexus injury	Iliac-ischiopubic fractures, sacral fracture	L5	10	Hemilaminectomy: neurolysis and decompression of S1 from a bone fragment	Global improvement of the leg

**Table 3:** Clinical evolution after conservative treatment

**N° of cases per injury patter**	**Root avulsions**	**Complete spontaneous recovery**	**Incomplete spontaneous recovery**	**No spontaneous recovery**
Lumbar plexus injuries: 5 patients*	**None**	**5**		
**Lumbosacral plexus injuries: 13 patients**	**None**	**7**	**6^**	
**Sacral plexus injuries: 18 patients**	**Avulsions were detected in 7 cases**	**5**	**4°**	**9**
**Complete LSP injuries: 6 patients**^**§**^	**Avulsions were detected in 3 cases**		**3**	**3§**
**Total number of patients: 42**		**17**	**13**	**12**

*1 bilateral lumbar plexus palsy.

^no recovery of foot dorsiflexion, in 4 cases posterior tibialis muscle scored M4 and these patients underwent a tibialis transfer.

°no recovery of foot dorsiflexion, tibialis tendon transfer was perfomed.

^§^1patient with an association of complete LSP injury and sacral plexus palsy, the latter presenting complete recovery.

### Does a correlation between the causative event and the injury pattern exist?

The overall analysis of our series demonstrated that a correlation between the causative event/mechanism and the injury pattern could be found; the associated bone injury was often predictive of the severity of the nerve injury.

In car crashes, after the impact occurred or the car overturned, the patient got trapped in and crushed, the trauma causing pelvic fractures: iliac-ischiopubic fractures, femoral fractures and sacral fractures were found in high percentage. In such cases, radiological studies frequently showed dislocated bone fragments compressing the endopelvic structures; in sacral fractures, the spinal roots were compressed by the fracture through the sacral foramina. Compression on the nerve structures resulted in injuries often amenable of spontaneous recovery.

Motorcycle accidents implied high kinetic energy traumas where traction did play an important role, as the high percentage of sacroiliac joint separations demonstrated (found in more than 50% of cases). In such cases lumbosacral plexus palsies were the result of severe, devastating stretch injuries: radiological studies always revealed root avulsions in patients with sacroiliac joint separations.

Concerning the clinical presentation, abolished sphincteral control was a bad prognostic factor as it was almost invariably associated with avulsions.

## Discussion

In recent times, reports have been published in the literature stating that LSP injuries might be significantly more frequent than it was thought in the past: Lindahl and Hirvensalo [9] report an incidence of LSP injuries equal to 40% in type-C pelvic ring injuries, Tonetti et al. [6] find out that about 52% of posterior osteo-ligamentary injuries were associated with neurological symptoms. The presumed rare incidence of these injuries might be due to the fact that they might go unrecognized in many cases [6,7,10]. Neurological complications are easily underestimated during the early assessment of these patients after the trauma. Patients are often multitraumatized and the life-treating injuries captivate the attention of the physicians; on the other hand the patient may be unconscious or uncooperative and thus unable to bring the palsy to notice. A careful, complete neurological examination is often difficult or impossible: Tonetti [6] reports that it could not be performed at admission in 10 out of 44 patients included in their series of posterior osteoligamentary lesions of the pelvic girdle.

Functional impairment of the lower limbs can also initially be put down to bone injuries. Weis [11] analysed 28 patients with a fracture of the acetabulum, pelvis or sacrum and found that in 11 cases there were electromyographic changes due to a lumbosacral plexus injury: clinical manifestations seemed very subtle but follow up demonstrated that even mild, subclinical functional impairment did interfere with the rehabilitation process.

In our series, only 30% of the nerve injuries were recognized during the early diagnostic assessment after the trauma, in many of the remaining cases there was a significant delay in diagnosis that resulted in late referral to the Authors. Moreover, the vast majority of these patients had been offered conservative treatment only. As previously pointed out, LSP injuries do recover spontaneously in high percentage whereas on the other hand surgery is known to be demanding and often resulting in poor outcomes: these facts nourish scepticism in many physicians that therefore advocate conservative treatment, ruling surgery out.

Yet the role of surgery should not be denied: although function in the foot cannot be restored after severe injuries, if reinnervation occurs in a few proximal key muscles of the leg the situation of the patient can dramatically improve, changing from being in a wheelchair to being able to stand and walk independently [7]. Surgery can also contribute in reducing pain, remarkably improving the quality of life and allowing to resume rehabilitation [7].

Better surgical outcomes could be obtained if early diagnostic assessment and a correct surgical timing were the rule: therefore it would be useful to establish precise guidelines of management and treatment.

Even if it might be objected that the number of patients in this series cannot account to statistically draw relevant conclusions, the present study clearly demonstrated correlations between the causative mechanism of the nerve injuries and their clinical presentation, pointing out possible indicators that seemed to distinguish the cases eligible for surgical treatment from those amenable of spontaneous recovery.

In plexus injuries, the first step of the diagnostic assessment is to detect the presence of root avulsions. When multiple avulsions are revealed, indication to surgery is undoubtedly straightforward: preganglionic injuries have no chances to recover spontaneously.

In this series root avulsions were never detected in lumbar plexus. These palsies were associated in high percentage to femoral fractures: the femoral nerve was injured by compression due to a peri-fracture hematoma and recovery was the rule, as it was previously found by Tonetti [6].

No root avulsions were revealed in lumbosacral trunk injuries; at follow up spontaneous recovery (complete or partial) always occurred, as it was also reported in the literature [6].

It might be therefore inferred that nerve surgery could be ruled out in these two injury patterns and rehabilitation is the treatment of choice (plus palliative surgery in case of incomplete recovery in lumbosacral trunk injuries).

Radiological studies revealed multiple root avulsions in sacral plexus and complete lumbosacral plexus injuries.

In our series root avulsions were invariably detected in patients with sacroiliac joint separations (found in high percentage in injuries sustained during motorcycle accidents), in agreement with findings previously reported in the literature [6,12].

Sacral fractures seem to mostly be associated to crush injuries in our series; in this kind of bone injury, a compression of the spinal roots by the fracture through the sacral foramina is reported between 22 and 56% of cases in the literature [6,13-16]. Although these cases are amenable of spontaneous recovery, experience has proved that early surgery with reduction of the fracture and consequent decompression of the spinal nerves leads to a faster and better recovery [6,14].

Thus we conclude that it is possible to extrapolate that in post-traumatic LPS injuries the clinical pattern itself can predict the occurrence of a spontaneous recovery and that the causative event does affect the injury prognosis. Patients that sustained the injury during motorcycle accidents have high chances to be candidates to surgery: in such traumas, traction does play an important role and root avulsions are likely to be found, especially if sacroiliac joint separation and loss of sphincterial control are associated with complete functional impairment. Severe, shooting leg pain is another bad prognostic indicator, as it was already pointed out in the literature [6,7].

Finally we would like to remark that in agreement with the literature [3-6,15,17,18], in our series spontaneous recovery occurred in high percentage of cases. A lower incidence of avulsions in comparison to the brachial counterpart (where avulsions can be found in up to 70% of cases) has been found in our series (23%) and in the literature [5,12,19] but it does not seem to be the only explanation. A high rate of anatomical variations of the lumbar and sacral roots have been reported in the literature [20,21], described as between 14 and 30% overall and 13% of L4 to sacral roots in cadaver studies: these seem to occur as extradural or intradural anastomosis as well as extradural nerve root divisions. Some of the recovery after a lumbosacral plexus injury can therefore occur through “collateral sprouting” from healthy or less severely injured neighboring nerves [7].

## Conclusions

Although the data reported in this study might need further confirmation in larger series, the present analysis demonstrated that in post-traumatic LSP palsies it is possible to predict the occurrence of spontaneous recovery based on clinical and radiological data. In lumbar plexus and lumbosacral trunk injuries spontaneous recovery always occurs and therefore rehabilitation is the treatment of choice. Sacral and lumbosacral plexus palsies also recover spontaneously in high percentage, when the nerve injury is due to compression by dislocated bone fragments: anyway in some cases (sacral fractures) surgical decompression can play a role allowing a faster and better recovery.

Palliative surgery can correct incomplete recovery.

Traction injuries (mostly occurring in motorcycle accidents) are more likely destined to severe, permanent disability. Patients with sacroiliac joint separations associated with a clinical presentation of complete functional impairment, loss of sphincteral control and shooting leg pain invariably reveal multiple root avulsions when accurate radiological investigations are performed: they should receive surgical treatment as soon as the general clinical conditions allow.

Early diagnostic assessment and referral to surgeons are certainly crucial to improve surgical outcome.

## Abbreviations

PT: Post-traumatic; LSP: Lumbosacral plexus.

## Competing interests

The authors declare that they have no competing interests.

## Authors’ contributions

DG conceived the idea of this study, analysed the data, drafted the manuscript, revised it and gave final approval of the version to be published. GZ and SF collected the data. All authors read and approved the final manuscript.
